# Microbiome and Breast Cancer: New Role for an Ancient Population

**DOI:** 10.3389/fonc.2020.00120

**Published:** 2020-02-12

**Authors:** Zahra Eslami-S, Keivan Majidzadeh-A, Sina Halvaei, Fatemeh Babapirali, Rezvan Esmaeili

**Affiliations:** ^1^Genetics Department, Breast Cancer Research Center, Motamed Cancer Institute, ACECR, Tehran, Iran; ^2^Laboratory of Rare Human Circulating Cells (LCCRH), University Medical Centre of Montpellier, Montpellier, France; ^3^University of Science and Culture, Basic Science and Advanced Technologies in Biology, Tehran, Iran

**Keywords:** estrogen metabolism, milk microbiome, microbiome chemotherapies, probiotic therapy, gene-based therapy, microbiome immunotherapy, microbiome radiotherapy

## Abstract

There are many risk factors associated with breast cancer (BC) such as the familial history of BC, using hormone replacement therapy, obesity, personal habits, and other clinical factors; however, not all BC cases are attributed to these risk factors. Recent researches show a correlation between patient microbiome and BC suggested as a new risk factor. The present review article aimed at evaluating the role of the microbiome as a risk factor in the occurrence of BC, investigating the proposed mechanisms of interaction between the microbiome and human genes involved in BC, and assessing the impact of the altered composition of breast, gut, and milk microbiome in the physiological status of normal breast as well as cancerous or non-cancerous breast lesions. The study also evaluated the growing evidence that these altered populations may hinder chemotherapeutic treatment. The role of microbiome in the development and maintenance of inflammation, estrogen metabolism, and epigenetic alterations was properly investigated. Finally, clinical and therapeutic applications of the microbiome- e.g., probiotics, microbiome genome modulation, and engineered microbiome enzymes in the management of BC were reviewed.

## Introduction

Breast cancer (BC) is the most common cancer among women worldwide. It includes Luminal A, Luminal B, Her2-enriched, and triple-negative subtypes based on the expression of estrogen, progesterone and Her2 receptors, and Ki67 protein (1). Genetic factors, hormone replacement therapy, lifestyle, eating habits, and age are among the BC risk factors (2); however, they cannot explain all the BC cases and other possible risk factors should be considered. In past decades, microbial composition of human body (microbiota) raised so much attention in different areas, including cancer biology. There is a dynamic and complex relationship between the human host and microbiota. Bacteria and their metabolites can manipulate different signaling pathways- e.g., E-Cadherin/β-catenin (3), cause DNA double-strand breaks (4), promote apoptosis, alter cell differentiation (5), and interact with toll-like receptors (TLRs) of the innate immune system to trigger inflammatory signaling pathways and help maintain the hemostasis of the body (6). The interaction between the human microbiome and cancer is referred to as “oncobiome” (7). Moreover, the human host can also affect the microbiota and their mechanisms (8).

Based on recent studies, the microbiome is a risk factor for BC and an explanation for different responses to therapy (9). The question that arises is “How can microbiome affect BC risk; in which mechanisms they detain or improve different therapeutic approaches; and what are the effects of probiotics on breast cancer management? “The disruption of commensal bacteria communities results in dysbiosis and may contribute to the development of carcinomas (10). For instance, it is observed that exposure to antibiotics (e.g., clarithromycin, metronidazole, and ciprofloxacin) decreases the biodiversity and abundance of some bacterial communities and disrupts the balance of the gut microbiome (11, 12) associated with a higher risk of BC (13).

Although it is not proved yet that dysbiosis can cause BC, the comparison of breast tissue samples show differences in the composition and abundance of some specific bacterial taxa between patients and healthy individuals (8). Therefore, there might be a relationship between breast microbiome and BC occurrence, which should be investigated.

There are fewer studies on microbiome and BC than other cancers. Particular composition of gut, breast tissue, and milk microbiome and the critical evidence of interaction with the inflammatory system, estrogen metabolism, and genetic and epigenetic alterations were also discussed in the current review. Moreover, the effects and mechanisms of microbiome on different therapeutic approaches such as hormone, chemo-, radio-, and immuno-therapy of BC were scrutinized. Although there are some reviews on microbiome and breast cancer (14–18), little is known about the clinical applications of the microbiome such as probiotics, microbiome genome modulation, and engineered microbiome enzymes in BC therapy, which also were discussed at the end.

## Microbiome and Bc: A Long Way to Find the Truth

### Breast Microbiome

It was believed that breast tissue is sterile, but now it is known that breast tissue has its specific microbiome, which is different from those of other tissue such as gut (8). Since breast is made up of fatty tissue with extensive vasculature and lymphatic drainage, it can be a favorable environment for the growth of bacteria (19). Early studies focusing on pathogenic viruses such as human papillomavirus (HPV) showed a correlation between HPV infection and BC (20, 21). In a study, about 32% of mammary tumors had an association with Epstein–Barr virus (EBV) or human herpesvirus-4 (HHV-4) infections (22). These results were controversial since no other studies confirmed them (23, 24). Investigations show a trilateral relationship between HPV infection, and signal transducer and activator of transcription 3 (STAT3) activity, and interleukin 17 (IL-17) level. HPV infection activates STAT3 signaling, which in turn raises IL-17 level. Therefore, HPV can be a factor in the development of pro-inflammatory responses in breast tissue and, hence, contribute to BC progression (25). Further studies are needed to prove the possible role of breast virome, the viral community in breast tissue, in maintaining the physiological status of breast tissue.

Next-generation sequencing services paved the way for finding the microbiome composition of the breast (26). The breast microbiome may be accumulated through different routes either during breastfeeding from the skin via the nipple-areolar by nipple-mouth contact, intercourse, or even through bacterial translocation from the gut (27). Local dysbiosis is observed in BC tissue compared to non-BC tissue. The analysis of published data sets with bioinformatics platform for microbial genomics revealed that the composition of microbiome community varies in patients with BC in different ethnicities; it also varies in nipple aspirate of survivors compared to those of the healthy individuals, is different in benign and malignant BC tissue, and varies in BC subtypes (17). To explain it, Urbaniak et al. identified eight new species and seven bacterial phyla in breast tissue samples. Accordingly, Proteobacteria and Firmicutes have a higher frequency that may be due to the adaptation to a higher amount of breast tissue fatty acid in comparison with other tissue (8). Some species in these groups of bacteria show a significant increase in BC samples. The results of clinical studies on the relationship between BC and microbiome are summarized in Table 1.

**Table 1:** Breast cancer microbiome from different studies.

**Sample size**	**Analyzed specimen**	**Identification assay**	**Finding**	**Discussion**	**References**
76 normal adjacent samples 5 healthy females	Breast tissue	16S rRNA V6	*Bacillus* sp., *Micrococcus luteus, Propionibacterium acnes*, *Propionibacterium granulosumm, Staphylococcus* sp., *Staphylococcus saprophyticus, Streptococcus oralis*, and *Streptococcus agalactiae* were the most abundant species in both the case and control tissue.	These species belong to Proteobacteria and Firmicutes families. Host microbial adaptation to the fatty acid environment in the tissue might be the reason for high prevalence of Proteobacteria and Firmicutes families. In comparison with healthy controls, *Escherichia coli* was significantly abundant in normal adjacent tissue, which its cancer-promoting activity is confirmed.	(8)
58 patients with BC and adjacent samples 23 healthy females	Breast tissue	16S rRNA V6	*Bacillus*, Enterobacteriaceae, and *Staphylococcus* sp., were more frequently found in cancerous samples. Microbiome profiles of normal adjacent and tumor tissue were almost the same.	By histone phosphorylation assay, it was shown that *Staphylococcus epidermidis* and *Escherichia coli* (belong to Enterobacteriaceae family) break double-stranded DNA of HeLa cells.	(28)
15 patients with BC and adjacent samples 13 benign breast lesion and normal adjacent samples	Breast tissue	16S rRNA V3–V5	In comparison with benign samples, *Fusobacterium, Atopobium, Gluconacetobacter, Hydrogenophaga*, and *Lactobacillus* genera were more frequently found in malignant samples.	*Fusobacterium* genus was significantly higher in malignant tissue samples. *Fusobacterium* genus may release factors and provide a pro-inflammatory environment, which leads to carcinogenesis.	(27)
100 females with BC 37 healthy females	Breast tissue (TNBC)	PathoChip array	In comparison with healthy samples, *Arcanobacterium, Brevundimonas, Sphingobacteria, Providencia, Prevotella, Brucella, Escherichia, Actinomyces, Mobiluncus, Propinibacteria, Geobacillus, Rothia, Peptinophilus, Capnocytophaga, Hepadnaviruses, Flaviviruses, Parapoxviruses, Herpesviruses, Retroviruses, Papillomaviruses, Pleistophora, Piedra, Foncecaea, Phialophora, Paecilomyces*/*Trichuris, Toxocara, Leishmania, Babesia*, and *Thelazia* were abundant in TNBC samples.	It is not identified if bacteria prepare the needed niche for promoting cancer, or tumor mass microenvironment prepares the needed niche for bacteria.	(29)
20 ER+ BC and their normal adjacent samples	Breast tissue	Pyrosequencing 16S V4 rDNA	*Proteobacteria, Firmicutes, Actinobacteria*, and *Bacteroidetes* were the most prevalent phyla in breast tissue. *Methylobacterium radiotolerans* was abundant in tumor tissue and *Sphingomonas yanoikuyae* in normal adjacent tissue.	The copy number of 16S rDNA, as an indication of bacterial amount, was not significantly different between normal adjacent tissue of BC patients and healthy individuals. The copy number of 16S rDNA was significantly lower in BC tissue.	(30)
25 females with a history of BC 23 healthy females	Nipple skin and nipple aspirate fluid	16S rRNA V4	In comparison with nipple aspirate fluid samples of healthy controls, *Alistipes* sp. was more prevalent in cancerous tissue, while Sphingomonadaceae had a higher prevalence in healthy samples.	*Alistipes* sp. was associated with colorectal cancer. Sphingomonadaceae family is known for its capability of decreasing aromatic hydrocarbons that are associated with BC.	(31)
57 females with invasive BC 21 healthy females	Breast tissue	16S rRNA V3-V4	*Methylobacteriaceae* significantly decreased; while *Corynebacterium, Staphylococcus, Actinomyces* genera as well as Propionibacteriaceae increased in patients with invasive cancer compared with healthy individuals.	Methylobacteriaceae producing phytohormones has an anticancer effect. Depletion of *Methylobacteriaceae* increases the cancer potential. On the other hand, *Corynebacterium, Staphylococcus* and *Actinomyces genera* as well as Propionibacteriaceae induce interferon-gamma (IFN-γ) secretion from T- and NK-cells, permit cancer cells to escape from T- and NK-cells recognition, and upregulate cell proliferation signals.	(32)
123 sentinel lymph node samples 123 normal adjacent samples	Sentinel lymph nodes and normal adjacent BC	RT-PCR and pyrosequencing	*Methylobacterium radiotolerance* abundance varied between lymph cancer nodes and normal tissue.	Microbial DNA may be involved in BC occurrence.	(33)
668 females with BC 72 normal adjacent samples	Breast tissue	16S V3–V5 RNA	*Actinobacteria, Proteobacteria*, and *Firmicutes* were the most abundant phyla in breast tissue. *Actinobacteria* sp. was abundant in adjacent non-cancerous tissue. *Proteobacteria* was abundant in tumor tissue.	*Mycobacterium fortuitum* and *Mycobacterium phlei* were differentially abundant in the breast tumor samples. Based on gene-set enrichment, *Listeria* spp. might be related to the expression profiles of genes associated with epithelial to mesenchymal transitions. *Haemophilus influenza* was related to the mitosis pathways: mitotic spindle assembly, E2F transcription factors, and G2M checkpoint.	(34)
148 females with BC 20 healthy females	Breast tissue	PathoChip array	*Proteobacteria, Firmicutes, Actinomyces* species were detected in each breast cancer type.	In each BC type, a unique viral, bacterial, fungal, and parasitic signature was observed. The distinct microbial signature was indicated in triple-negative and -positive samples. In contrast, a similar microbial pattern was identified in the ER- and HER2-positive samples.	(35)
21 BC and their normal adjacent samples	Fresh breast tissue	Hypervariable region of the 16S-rRNA gene (V3)	*Proteobacteria, Firmicutes, Actinobacteria*, and *Bacteroidetes* were, respectively the most abundant phyla in breast tissue. The abundance of *Methylobacterium* varied among patients.	Slight differences were detected between critical microbiome composition of tumors and adjacent normal tissue. Major differences were detected between cancerous and healthy samples.	(36)
22 females with benign breast lesions 72 patients with invasive BC	Breast tissue	16S V1-V2 rRNA	*Propionicimonas* genus as well as *Micrococcaceae, Caulobacteraceae, Rhodobacteraceae, Nocardioidaceae*, and *Methylobacteriaceae* families was abundant in malignant tissue.	As malignancy is developed, the prevalence of Bacteroidaceae family decreases, and the relative abundance of *Agrococcus* genus (Microbacteriaceae family) increases. Compared to grade 1 and 2 tumors, in grade 3 tumors, glycerophospholipid metabolism and ribosome biogenesis pathways were upregulated, and flavonoid biosynthesis significantly decreased in grade 3 tumors.	(37)
60 healthy postmenopausal females	Urine and fecal samples	Pyrosequencing of the V1–V2 region of 16S rRNA	The ratio of estrogen metabolites to parent estrogen was directly associated with the abundances of several taxa in the Clostridia class. Inversely, *Bacteroides* genus had a negative correlation with this ratio.	Patterns of estrogen metabolism were associated with the diversity of the gut microbiome.	(38)
32 females with BC	Fecal samples	PCR targeting 16S rRNA	Compared to females with grade 1, total number of *Blautia* sp., increased in females with grade 3. Based on BMI, significant differences were observed in the whole number bacteria and certain bacterial groups (*Egerthella, Blautia, Firmicutes*, and *F. prausnitzii*).	Based on clinical stages, total numbers of *Bifidobacterium* and *Blautia* species and quantity of *F. Prausnitzii* and *Blautia* sp. were significantly different.	(39)
48 postmenopausal females with BC 48 healthy females	Urine and fecal samples	Illumina sequencing and taxonomy	Elevated levels of *Clostridiaceae, Faecalibacterium*, and *Ruminococcaceae* were detected in patients with BC. In contrast, levels of *Dorea* and *Lachnospiraceae* decreased in patients with BC.	Fecal microbiota composition altered in postmenopausal females with BC.	(40)
31 females with BC	Fecal samples	qPCR targeting 16S rRNA	The total number of *Firmicutes, Faecalibacterium prausnitzii, Blautia* sp., and *Eggerthella lenta* was significantly higher in overweight patients.	Microbiome composition differed based on BMI.	(41)
48 postmenopausal females with BC	Urine and fecal samples	16S V4 rRNA	Alpha diversity significantly reduced in patients with BC. Composition of both IgA^+^ and IgA^−^ fecal microbiota was also altered in patients with BC.	Significant estrogen-independent, related to the IgA^+^ and IgA^−^ gut microbiota was detected in patients with BC.	(42)
18 premenopausal females with BC 25 premenopausal healthy females 44 postmenopausal females with BC 46 postmenopausal healthy females	Fecal samples	Illumina sequencing	*Escherichia coli, Citrobacter koseri, Acinetobacter radioresistens, Enterococcus gallinarum, Shewanella putrefaciens, Erwinia amylovora*, and *Actinomyces* sp., were the most prevalent species in postmenopausal patients with BC. In postmenopausal BC samples, *HPA0247, Salmonella enterica, Fusobacterium nucleatum, Eubacterium eligens*, and *Roseburia inulinivorans* were less frequent.	Composition of the gut microbiome varied in postmenopausal patients with BC and healthy females, but had no significant difference with that of the premenopausal controls.	
48 postmenopausal females with BC 48 postmenopausal healthy females	Fecal DNA samples	qPCR (primers were designed for the known baiH ORF in different bacteria)	BaiH of *Clostridium sordelli, Pseudomonas putida*, and *Staphylococcus aureus* had lower abundance in patients with BC. The sharp abundance of the baiHof *Bacteroides thetaiotaomicron* and *Pseudomonas putida* was noticed in patients with early-stage BC.	BaiH ORF in bacterial species had different abundance between patients with BC and healthy individuals.	(43)
32 females with low-stage BC	Fecal samples	16S V4 rRNA	In females with higher body fat, *Akkermansia muciniphila* (AM) number was lower. In females with HAM (high number of AM), alpha diversity was more elevated. In patients with LAM (low number of AM), *Prevotella* and *Lactobacillus* genera were more frequent, while the number of *Clostridium, Campylobacter*, and *Helicobacter* was lower.	In individuals with early-stage BC, body composition was related to microbiome diversity, AM, and IL-6 level.	(44)
48 postmenopausal females with BC 48 postmenopausal healthy females	Fecal DNA samples	qPCR (primers were designed for known CadA and LdcC genes in different bacteria)	In patients with BC, *Escherichia coli* CadA, *Escherichia coli, Enterobacter cloacae*, and *Hafniaalvei* LdcC DNAs were less frequent. Compared to all patients, CadA and LdcC abundance decreased in patients with stage 0 cancer. Compared with healthy females, *Escherichia coli* LdcC protein levels were markedly lower in the fecal samples of patients with stage 1 cancer.	Compared with healthy females, the DNA coding LdcC and CadA had a different abundance in bacterial species of patients with BC.	(45)

Although some studies showed no evidence of bacterial abundance in BC (30), other investigations indicated that diversity (40) and abundance (41) of associated taxa are reduced in BC. The analysis of 16S rRNA showed a higher relative abundance of Enterobacteriaceae, *Bacillus* and *Staphylococcus* spp. in patients with BC. Interestingly, *Escherichia coli* (Enterobacteriaceae family) and *Staphylococcus epidermidis* induce double-stranded DNA break in BC cells (28).

**Figure 1:**
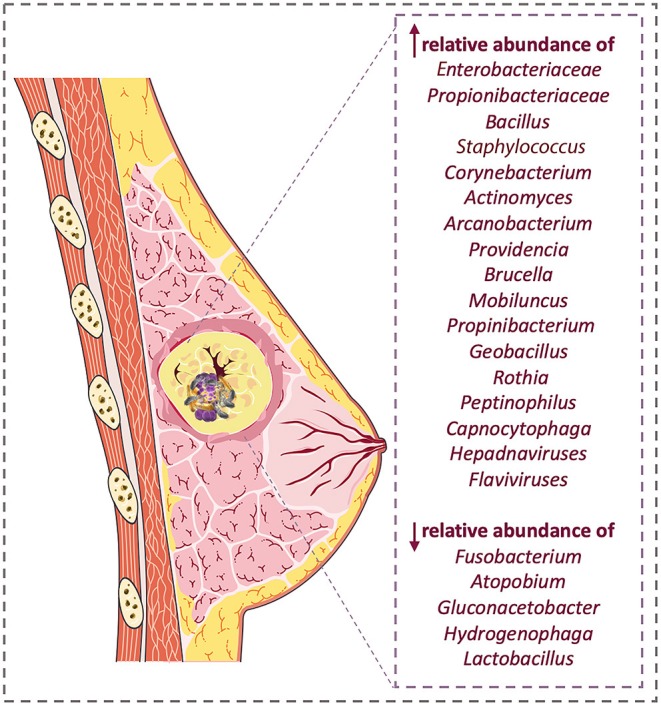
Breast cancer microbiome change; there are significant differences in microbiome population in patients compared with healthy samples.

There is a significant difference in the microbiome combination of malignant and benign breast tissue specimens (27) (Figure 1). An overview of taxonomic profiles showed that the overall microbiome of breast tissue was similar in benign and invasive BC, dominated by *Bacteroidetes* and *Firmicutes*. Assessing differential taxa between these two groups demonstrated malignancy correlated with enrichment in taxa of lower abundance, including the genera *Fusobacterium, Atopobium, Gluconacetobacte*r, *Hydrogenophaga*, and *Lactobacillus* (27). The carcinogenicity of 10 infectious pathogens is proven (46). Therefore, a greater understanding of the effects of microbial agents within BC can expand the ability to prevent, diagnose, and treat it in the future. In this regard, studying viral, bacterial, fungal, and parasitic genomic sequences led to the discovery of two distinct microbial signatures in patients with triple-negative BC (TNBC) (29). However, further studies are needed to identify how these signatures affect BC development. Nevertheless, there is a large amount of evidence that microorganisms regulate the tumor microenvironment. Finding a unique microbial signature for TNBC may have diagnostic and targeted therapeutic applications in the future (29).

Quantification of bacterial DNA in BC showed that *Methylobacterium radiotolerance* was abundant in tumor tissue, while *Sphingomonas yanoikuyae* (*S. yanoikuyae*) was dominant in the normal adjacent tissue samples (30). Additionally, the amount of bacterial DNA significantly reduced in tumor tissue compared to the adjacent tissue. The association of *S. yanoikuyae* with healthy tissue and its significant reduction in the tumor tissue might be a confirmation of the probiotic function of this microorganism in the breast. The lower amount of *S. yanoikuyae* in tumor areas led to reducing one-third of antibacterial gene expression responses. Innate immune system receptors such as TLRs 2, 5, 9, and the factors responsible for anti-microbial responses such as IL-12 subunit alpha (IL-12A), bactericidal/permeability-increasing protein (BPI), and myeloperoxidase (MPO) were expressed minor in the tumor in comparison with healthy tissue.

The difference in breast tissue microbiome profile between healthy individuals and patients with BC was also confirmed in another study (20). This difference was also observed in patients with BC at various clinical stages (39). There was a clinical trial (MICROMA) (NCT03885648) that evaluated contribution of bacteria, archaea, viruses, and fungi in breast tissue, stool, and urine samples with their alteration by environmental contaminants to the risk of BC. The results could contribute to elucidate risk factors, improve the prognosis, and propose new intervention studies in BC.

This evidence suggests that bacteria may maintain the healthy status of breast tissue by stimulating host inflammatory responses. Reduction of bacterial load in a healthy individual may exacerbate the risk of BC. These findings demonstrated an unknown link between dysbiosis and BC, and the potential diagnostic and therapeutic implications of these discoveries should be investigated in further investigations. Although many studies were performed on the body-wide impacts of microbiota, there is still no clear pattern about the direct effect of these microorganisms on the risk of BC.

### Milk Microbiome

Findings of the bacterial biodiversity in human breast milk and its changes over time are limited. However, culture-independent molecular techniques such as qPCR and NGS approaches allow valuable complementary assessments of the human milk microbiota.

Pregnancy, childbirth, postpartum period, diet, and consumption of antibiotics are key factors that affect the bacterial biodiversity of human milk. The bacteria of human milk originate from the gastrointestinal tract and are transferred to the breast through the entero-mammary path. They can also be transmitted from the infant's mouth via maternal skin contact during breastfeeding (47). Breast and milk microbiomes are almost similar. The most abundant phyla of both breast tissue and breast milk are *Firmicutes, Actinobacteria*, and *Bacteroidetes* (36). It is observed that frequent bacterial strains, including *Staphylococcus, Serratia, Corynebacteria*, and *Streptococcus* are the most abundant bacteria in milk (48). In the studies on human milk, various species of *Bifidobacterium* are reported (49).

Furthermore, several cohort studies are conducted based on geographical variations (50–52) demonstrating that the microbial community is significantly variable in different geographic locations. For instance, the highest relative abundance of *Proteobacteria, Firmicutes, Streptococcus, Propionibacterium*, and *Pseudomonas* were in South African, Finnish, Chinese, and Spanish human breast milk samples, respectively. It is assumed that geographical distinction creates remarkable changes in the composition of the microbiome (50).

Milk contains more than 360 genera of prokaryotes including phyla of *Proteobacteria* (65%), *Firmicutes* (34%), and the genera of *Pseudomonas* (61.1%), *Aureus* (33.4%), and *Streptococcus* (0.5%). Milk has less biodiversity in comparison with infants' and mothers' stools at the level of phylum. Researches show that enriched immune-modulatory DNA motifs of *Lactobacillus* contribute to immune development by modulating the immune responses (53). Moreover, studies by Ward et al. on the functional capacity of milk metagenome showed that these sequences along with open reading frames associated with nitrogen metabolism, membrane transport, and stress response in the human intestine resulted in the colonization of the newborn's gut and immunity. Given the presence of immune-modulatory motifs in the milk metagenome, further investigations on this biological fluid are warranted (54). Some of these motifs in commensal bacteria can hypothetically be used for therapeutic purposes.

Understanding the functions and composition of the human milk microbiota has critical implications in terms of the infant gut microbiome establishment and the mammary health since dysbiosis in the milk microbiome may prime to mastitis (55). Metagenomic analysis indicated that *Firmicutes, Proteobacteria*, and *Bacteroidetes* were not found in milk samples of patients with mastitis when compared to samples from healthy individuals (56). However, *S. aureus* and *S. epidermidis* were abundant in patients with acute and subacute mastitis, respectively.

In contrast, *Staphylococcus, Streptococcus, Bacterioides, Faecalibacterium, Ruminococcus, Lactobacillus*, and *Propionibacterium* species were isolated from samples obtained from healthy individuals (56). Lower microbial diversity, depletion of commensal obligate anaerobes, and increased abundance of opportunistic pathogens in patients with mastitis were confirmed in another study. Functional metagenomics identified several gene pathways in bacterial secretion system and motility proteins related to bacterial proliferation and colonization in sub-acute and acute mastitis samples. It was reported that ~45% of genes belonged to metabolism, 18% to environmental information and processing, 14% to genetic information processing, and 1% to human diseases (57).

A significant difference in the microbiome composition of nipple aspirate fluid between healthy individuals and patients with BC suggested the potential role of the ductal microbiome in BC incidence. The analysis of nipple aspirate fluid in patients with BC revealed a relatively high proportion of *Alistipes* genus, while an unclassified strain of the Sphingomonadaceae family was more abundant in healthy individuals. Moreover, BC-related microbes increase beta-glucuronidase activity that may increase cancer risk (31). It is also proved that chemotherapy results in significant deviations of healthy microbial populations and their metabolomic profiles. A decrease in *Bifidobacterium, Eubacterium, Aureus*, and *Clobacterium* species and an increase in *Acinetobacter, Xanthomonadaceae*, and *Stenotropomonas* species were observed in the milk of healthy females and those of the ones undergoing chemotherapeutic treatment for Hodgkin's lymphoma (58).

### Gut Microbiome

#### Host-Gut Microbiome Interaction

The human gastrointestinal tract supports the growth of beneficial microbiota owing to their ability to protect the body against pathogens (59). Their contribution to immune system development and maintenance (60), the fermentation of indigestible fibers into short-chain fatty acids (SCFAs) (61), production of essential amino acids (62) and vitamins (63), absorption of minerals (64), and deactivation of toxins (65) and carcinogens (66) are among their benefits. Animal model studies showed associations between the microbiome and the development of many diseases, including cancer (67, 68). Diet can contribute to the development of various diseases, including cancer (69), since it has a direct role in controlling the composition of the microbial community. Accordingly, a plant-based diet stimulates bacterial diversity (70), while animal-based regimen decreases the *Firmicutes* population (the common bacterial phylum in breast tissue) in digestive system. Shifting to a plant-based diet would increase *Firmicutes* population (71). Thus, changes to the diet might contribute to the development of diseases through alternation in microbial metabolism and production of toxic metabolites.

Some evidence shows that the microbiome metabolite influences the occurrence of BC; this hypothesis may help estimate the cancer risk and prevention. For instance, cadaverine as a biogenic amine is formed through the direct decarboxylation of L-lysine (45). It is reported that cadaverine biosynthesis is reduced in the gut in early-stage BC, resulting in lower production of an anti-cancer bacterial metabolite and reduced BC invasion (45). It is shown that bacterial metabolites can stimulate oxidative and nitrosative stress that inhibit BC progression. These metabolites such as lithocholic acid can inhibit BC progression, epithelial-mesenchymal transition, and metastasis via activation of nuclear factor erythroid 2-related factor 2 (NRF2) and other proteins involved in the antioxidant defense system. Therefore, decreased microbiome diversity and quantity in gut microbiota affects these anti-proliferative metabolites that may result in BC or its progression (43). As previously mentioned, SCFAs are produced by microbiome when dietary fiber is fermented in the colon (72). Propionate, acetate, and butyrate are the three most predominant SCFAs. They are well-known modulators for cell invasion and apoptosis in BC (73). SCFAs can positively or negatively affect BC (74). The abundance of *Akkermansia muciniphila*, as a key player of propionate production, is associated with the richness of the gut microbiota in patients with BC (44).

It was shown that intestinal bacteria can turn some plant lignans such as flaxseed, sunflower, caraway, pumpkin, legumes, and soybean (75), into mammalian lignans with protective effects against BC (76–78). Lignans of edible plants are converted to enterolignans, enterolactone, and enterodiol by the intestinal microbiome. It is suggested that enterolactone may act as a selective modulator of estrogen signaling and may be associated with lowering the risk of BC (79, 80).

Additionally, lignans consumption may both enhance the survival of postmenopausal female patients with BC (81) and reduce the risk of BC before menopause (82). A significant inverse correlation was observed with BC risk in premenopausal females daily receiving ≥30 g of fiber, fruits, or seeds (83). Also, high consumption of raw vegetables showed a significant protective effect against BC risk; being dropped by 34% (84). It was observed that high levels of plant dietary fibers in the gut resulted in proliferation of *Bifidobacterium* and *Faecalibacterium prausnitzii* (85) with anti-inflammatory (86, 87) and anti-tumor effects (88). Studies on twins represent that obesity alters the balance of Firmicutes in non-obese individuals to Bacteroidetes phyla in obese ones (89). This shift may result in the increase of estrogen levels in the blood and contribute to higher risk of BC. Diet is the main element of gut microbial diversity. But, recent studies on non-human primates show the effect of Mediterranean diet on increasing mammary gland *Lactobacillus* abundance and upper levels of bile acid metabolites (90).

Therefore, it can be concluded that modifying dietary patterns affects the microbiome population and indirectly affects disease occurrence (91).

#### Gut Microbiome Dysbiosis

Using antibiotics can affect the target pathogen and the commensal inhabitants of the human host. The extent of the impact on non-target microbial populations depends on the particular antibiotic used, its mode of action, and the degree of resistance in the community. Bhatt et al. found that irregular use of antibiotics increases the probability of dysbiosis and lower bacterial diversity (92). Knekt et al. showed that the overuse of antibiotics might reduce the plasma level of lignan enterolactone; therefore, it might directly affect the microbiome populations and increase the BC risk (93). However, it is acknowledged that selection of antibiotics leads to antimicrobial resistance, but it is believed that the commensal microbiota is normalized a few weeks after treatment secession (11).

In comparison with other human organs, the microbial load and its variety are increased in the digestive system, especially in the large intestine (94). This complex intestinal microbiome plays a significant role in both local and distal areas of the body through the production of metabolites, hormonal intermediates, and immunologic cytokines (95). It was shown that higher phylogenetic diversity in the intestinal microbiome raises hydroxylated estrogen metabolites in the urine of a healthy female (96). In postmenopausal females, an increased level of circulating estrogen is associated with increased risk of BC (97). Intestinal microbiome is one of the major regulators of circulating estrogens (98). Therefore, dysbiosis in the gut microbiome potentially disrupts homeostasis through the disruption of estrogen metabolism (98). It is suggested that estrobolome, the bacterial gene mass in the human intestine, the products of which take part in estrogens metabolism, may increase the risk of estrogen receptor-positive BC in postmenopausal females (99, 100). Additionally, it was shown that a decrease in stool bacterial biodiversity leads to estrogen excretion and finally elevation of the BC risk (101). In contrast, an increase in the levels of estrogen metabolites, compared to parent estrogens, estrone, and estradiol, decreases the risk of BC (102).

Postmenopausal females recently received BC diagnosis, had higher levels of urinary estrogen, which does not correlate with their bacterial biodiversity (40). Compared to controls, postmenopausal patients with BC had significant estrogen-independent associations with the IgA^+^/IgA^−^ gut microbiota, suggesting that the gut microbiome may influence the BC risk by altered metabolism, estrogen recycling, and immune pathway (42).

Interestingly, intestinal bacteria are capable of converting plant lignans into enterolignans (103), which can induce estrogenic effect (104). Furthermore, phytoestrogen can start signaling through estrogen receptors when present at extremely high levels, reducing the activity of natural estrogen hormones in the body and blocking the effect of estrogen in specific tissue (105, 106). Therefore, changes in gut microbiome composition may lead to the estrogen metabolism alternation and affect the BC risk.

The microbiome might also diminish the risk of BC by modulating the functional estrogen. The current hormone replacement therapies might be further enhanced by combining the microbiota, which acts individually or synergistically to improve a more comprehensive therapeutic approach for metabolic diseases after menopause (107).

Notably, it is found that gut microbiome diversity might contribute to better psychosocial and cardiorespiratory fitness outcomes in the post-primary treatment of BC survivors. Microbial profiling of fecal samples showed that prospective alteration in the gut microbiome was significantly associated with depression, anxiety, fatigue, and cardiorespiratory fitness outcomes related to the quality of life of the patients. The extent of longitudinal changes in fatigue, anxiety, and cardiorespiratory fitness was correlated with the frequency of the *Prevotella, Faecalibacterium, Bacteroides*, and *Coprococcus* genera, a subset of the Clostridiaceae family, and *SMB53* and *Roseburia* genera (108).

Bacterial populations employ molecular signals to communicate with each other (109, 110). Bacteria can communicate with ecological conditions, detect environmental changes, or sense the abundance and type of living bacterial species via chemical communications called quorum sensing (111). De Spiegeleer et al. proved that many pathogenic and commensal bacterial species produce quorum sensing compounds in the human intestine. These endogenous compounds including phosphatase RapG inhibitor (PhrG) produced by *Bacillus subtilis*, competence stimulating peptide (CSP) produced by *Streptococcus mitis*, and extracellular death factor (EDF) produced by *E. coli*, cause *in vitro* angiogenesis and BC cells invasion (112).

Dysbiosis of the microbial population often results in the disruption of the host immune system. Changes in the microbial community lead to lymphocytes decrease, and neutrophils increase, both of which can contribute to a reduction in survival of patients with BC (107). Studies on mice specimens showed that gut microbiome alterations lead to breast tumorigenesis (113). There is an ongoing clinical trial (NCT02696759) that investigates whether the gut microbiome plays a role in fighting advanced BC by affecting the efficacy of immune cells.

According to another study, the proportions of *Blautia* and *F. Prausnitzii* and absolute numbers of *Blautia* and *Bifidobacterium* species in the gut microbiome are directly correlated with the clinical stage of BC. For instance, patients with stage 1 BC had a lower number of gut *Blautia* sp. in comparison with the ones with stage grade 3 (41). The presented shreds of evidence showed that the gut microbiome plays a fundamental role in the development of various diseases, including cancer. Therefore, therapeutic targeting of the gut microbiome should be explored as part of the preventative and therapeutic approaches.

## Clinical Targeting of the Microbiome: A Fairytale or Sensible Approach?

### Probiotic Therapies

**Figure 2:**
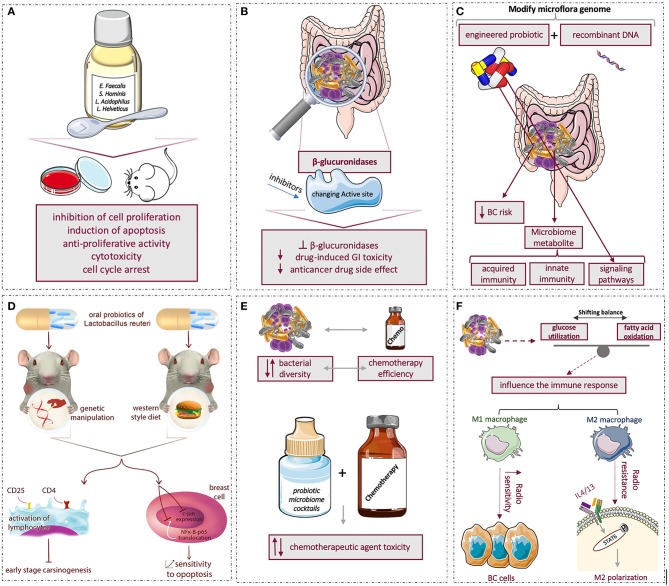
Summary of microbiome effects and applications in breast cancer: **(A)** using probiotics to affect tumor progression by inhibiting cell proliferation and inducing apoptosis **(B)** conformational changes in β-glucoronidaze enzyme active site and different catalytic activities with genetic engineering might serve as targets to decrease the anticancer drug-induced toxicity, **(C)** using novel engineered recombinant probiotic to modify and target gut microbiome to reduce breast cancer risk, **(D)** The left-side mouse model was genetically manipulated to develop human breast tumor while right-side mouse model was fed by the Western-style diet to develop mammary tumors. Both models were treated with oral intake of probiotic lactic acid microbe, *Lactobacillus reutri*. The investigation showed that oral probiotic activates CD4^+^ and CD25^+^ lymphocytes and inhibits early-stage breast carcinogenesis. Moreover, oral probiotic prevented c-jun expression and NFκ-B-p65 translocation in the nucleus of breast cells and raised breast cell sensitivity to apoptosis, **(E)** reciprocal interaction of chemotherapy with bacterial diversity; using combination of chemotherapy and probiotics microbiome cocktails showed no effect, decreased and in some cases increased the chemotherapy agent toxicity, **(F)** microbiome by shifting balance of glucose utilization and fatty acid oxidation can indirectly affect immune system; hence, during radiotherapy, M1 macrophages increase the radio-sensitivity of BC cells, but M2 macrophages trigger radio-resistance via IL-4/IL-13-mediated STAT6 phosphorylation and M2 polarization.

Correlations between the human microbiome and BC open up new horizons for the prognosis and treatment of cancer. Hence, researchers focus on the therapeutic application of microbiome (Figure 2).

Several *in vitro* and *in vivo* studies investigated the effect of probiotics on BC; for instance, significant inhibition of cell proliferation, induction of apoptosis, and cell cycle arrest of *Enterococcus faecalis* and *Staphylococcus hominis* are proved (114). Lakritz et al. studied two groups of mice: a group manipulated to develop human breast tumors and the other group fed by a Western-style diet (high fat and sugar, low vitamin D3, vitamin C, and fiber) to develop mammary tumors. The two groups were treated with oral intake of probiotic lactic acid microbes. The results showed that the probiotic *Lactobacillus reuteri* inhibited early-stage carcinogenesis and raised breast cell sensitivity to apoptosis (115).

Additionally, it was confirmed that oral administration of *L. acidophilus* represents anticancer activity in mice bearing breast tumors (116). Another *in vivo* study showed that drinking milk fermented with *Lactobacillus helveticus R389* elevated IL-10 and decreased IL-6 levels both in serum and mammary cells of mice, which lead to breast tumor cell inhibition (117). Moreover, anticancer effects of probiotics on cancer cell lines are well gathered in the review by Mendoza et al. They showed anti-proliferative activity, apoptosis, cytotoxicity, and cell cycle arrest of probiotics (118). Long-term exposure to probiotics such as *L. casei Shirota* and soy isoflavones in Japanese females demonstrated their chemopreventive effect on cancer development (119).

Although the mentioned studies provided the evidence that probiotics display activity against BC, there are still essential questions on the use of probiotics in BC. The strains, dosage, and regimen of probiotics should be determined based on the clinical feature of BC and probiotics interaction with the conventional treatment. Probiotics are already used in the treatment of a wide range of diseases; however, their application to BC is in its infancy. There are also some clinical trials on probiotics and BC. A study demonstrated that two species of *Lactobacillus* can treat mastitis (120). Thus, probiotics might be good alternatives for antibiotics to treat breast infections during breastfeeding (120).

In the (NCT03358511) clinical trial, the role of probiotics on the number of CD8^+^ T-cells at stages 1–3 BC in post-menopausal patients is under investigation. Twenty post-menopausal females with BC took probiotics (15 billion colony-forming units of 13 beneficial bacterial species) for 2–4 weeks, three times a day. Another trial (NCT03760653) determined the effect of probiotics supplementation (*Lactobacillus rhamnosus, Lactobacillus paracasei, Lactobacillus acidophilus, and Bifidobacterium bifidum*) and physical exercise on the bacterial balance of gut, immune system, and the quality of life in BC survivors.

Although using probiotics inhibited tumor growth, induced apoptosis, and also enhanced the immune system in *in vitro* and *in vivo* studies, using them as a promised treatment in the clinical practice is not established yet. Although not confirmed, it seems that the tissue-specific microbiome cannot be changed even with long-term applications of probiotics; hence, the aforementioned positive effects might be obtained by modulating the gut microbiome and subsequently preventing tumorigenesis in the breast.

### Gene-Based Therapies

TLRs are deregulated in some types of cancers and can drive a pathological immune activation in response to the normal microbial mass (121). For instance, it is suggested that activation of TLR5 by *S. typhimurium* flagellin in BC cells mediates the pro-inflammatory responses to obtain an effective anti-tumor activity, which may serve as a novel therapeutic target for BC therapy (122). Successful inhibition of this pathway would be a unique therapeutic avenue worth exploring.

Another suitable therapeutic approach is the potential of the bacterial β-glucuronidases enzyme (123, 124) released by the gut microbiome, and is required for the healthy digestive system and xenobiotic metabolism (125, 126). Small changes in the structure of inhibitors can cause particular conformational changes in the enzyme active site and different catalytic activities, which might serve as targets to decrease the anticancer drug-induced toxicity in the intestinal tract; in other words, they can reduce the side effects of anti-breast cancer drugs (123). As this enzyme has substantial roles, beneficial engineered bacteria with modified proteins might be therapeutically used to minimize drug side effects. This area needs more detailed information at basic and clinical levels to confirm the positive effects of the microbiome and its metabolites on gene-based therapy in patients with cancer.

### Microbe-Chemo Therapies

The microbiome affects chemo-, immuno-, and radio-therapy for BC. In the absence of commensal microbes, the activity of platinum-based drugs is diminished, although they enter the cells. DNA damage and double-strand breaks are essential in platins. The production of ROS promotes these mechanisms via microbes (127). For example, *Lactobacillus acidophilus* can restore the cisplatin antitumor activity in germ-free mice (9).

There is clinical evidence of immunotherapy showing better survival in triple-negative BC with anti-PD1 antibody (128). Previous studies show that antibiotic therapy for lung cancer and, accordingly, reduction of *Akkermansia muciniphila* would diminish the effect of anti-PD1 antibody and reduce the survival time in patients. It is hypothesized that T-cell mediated response is stimulated by IL-12, and the increase of anti-tumor activity of cytotoxic T-cells in response to *A. muciniphila* may improve the clinical response to this antibody (129). The same mechanism can be extended in BC, while antibiotics are often prescribed to females with BC during or after surgery.

There are limited studies on microbiome and radio-response in BC. Previous studies showed that cancer radiotherapy has less effect on germ-free mice compared to intact mice (130). It can be concluded that antibiotic therapy may reduce the effect of cancer radiotherapy. Intestinal bacteria and fungi can alter the immune system in determining the response to radiation. It was shown that during macrophage polarization, the metabolic situation of the cell would be changed (131). Radiotherapy response or resistance is highly dependent on tumor microenvironment (TME). The byproducts of the microbiome alter the metabolic situation by shifting the balance between glucose utilization and fatty acid oxidation, influencing the immune response in the TME (132). This alteration may change radio-sensitivity of the cancer cells. M1 macrophages enhance the radio-sensitivity of BC cells, but M2 macrophages trigger radio-resistance via IL-4/IL-13-mediated STAT6 phosphorylation and M2 polarization (132, 133).

While chemotherapy can change the bacterial diversity, specific microbiome composition can, in turn, modify the efficacy of chemotherapy. Therefore, it is feasible that specific probiotic microbiome cocktails can be administered in combination with chemotherapeutic agents to enhance their functionality (92). Lehouritis et al. examined the effects of bacteria through their enzymatic regulation on 30 common chemotherapy medications *in vitro*. They revealed that wild-type bacteria might increase the toxicity of six chemotherapeutic agents, decrease the toxicity of nine others (including doxorubicin), and not affect the toxicity of the rest 15 drugs. Thus, the response to therapy in BC tumors may be improved by microbiome modulation (134). Local and systemic bacterial infections act as *in situ* bio-transforming reservoirs. They influence treatment efficacy and increase the toxicity outside the targeted area that may complicate the cancer treatment process (134).

To date, few studies addressed the link between the gut microbiome and BC chemotherapy. NCT03586297 demonstrates the associated dominance of specific gut and intratumoral microbiome with the pathologic response in newly diagnosed patients with TNBC receiving AC-T neoadjuvant chemotherapy. Another trial (NCT04138979) recruited 80 participants to explore more information about the intestinal microbiome of patients with BC undergoing chemotherapy. These studies can prove that gut microbiome analysis holds the potential to predict patient response to chemotherapy before treatment and personalize medicine. However, further studies are required to understand the biochemical interactions between therapeutic agents and bacteria. In addition, new combinations of chemotherapy with probiotic treatments should be taken into consideration.

### Antibiotics

Microbiome engineering may open new horizons in prevention, diagnosis, and treatment of cancer. As mentioned above, alterations in gut bacterial populations may increase the risk of cancer. Therefore, designing antibiotics that target a particular spectrum of the microbiome might help regulate the gastrointestinal microbiome as a possible way to reduce the BC risk (135). Since cancer occurrence is affected by suppression of the immune system and many proinflammatory pathways (136), it is not surprising that the microbiome and bacterial metabolites might have a direct effect on the incidence or progression of cancer (137, 138). It may occur through changes in the activation of signaling pathways (139) as well as the innate and acquired immune responses (60, 140).

Consequently, engineered probiotics might be useful in targeting these signaling pathways. The combination of microbiome engineering and recombinant DNA technology can be utilized to modify the genome of vital microflora compartments and reprogram microbial mechanisms (141). The future discovery of dominant or unique members of the microbial population has the potential to drive new ideas in bacteriotherapy (142–145).

Microbiome diversity across different people caused by pathological, physiological, and environmental differences is a crucial challenge when trying to define beneficial or pathological microbial signatures. The complex diversity of these microorganisms makes it challenging to identify specific cancer signatures that are stable over time.

## Conclusion

The microbiota plays a crucial role in preserving the health status of the human body, and their impairment causes pathobiological changes, including BC. Although the evidence of the correlation of microbiome with BC is undeniable, there are essential questions to unlock the exact role of the microbiome in the development and treatment of BC. Strains, dosage, and regimen of probiotics based on the clinical feature of BC and probiotics interaction with the conventional treatment are not determined entirely yet. Further studies are needed to find the exact relationship between microbiome and cancer. In other words, it needs to be clarified whether microbiome alteration leads to cancer or cancer occurrence leads to microbiome alteration, whether dysbiosis is carcinogenic or if there is a way to regulate dysbiosis. To answer these questions, large-scale studies including animal models, retrospective and prospective ones, as well as clinical trials should be designed. Engineered bacteria such as probiotic products might be new modalities to develop a therapeutic approach on clinical scale.

## Author's Note

This manuscript was originally submitted to the Cancer Metabolism section of Frontiers in Oncology.

## Author Contributions

RE and KM-A: conceptualization. RE, ZE-S, SH, and FB: drafting of the manuscript. RE, SH, and ZE-S: designing figures and tables. RE, SH, ZE-S, and KM-A: review and editing of the manuscript. All authors read and approved the final manuscript.

### Conflict of Interest

The authors declare that the research was conducted in the absence of any commercial or financial relationships that could be construed as a potential conflict of interest.
